# Alas, Poor Yorick: Digging Up the Dead to Make Medical Diagnoses

**DOI:** 10.1371/journal.pmed.0020060

**Published:** 2005-03-29

**Authors:** Deborah Hayden

## Abstract

Is it ethical to dig up famous dead people to make tissue diagnoses?

## Teaser

Exhuming famous dead people to test their tissues is mired in legal, ethical, and moral problems

## Preparing for Death

How does one prepare for death? Those who have created a public persona must add to any spiritual ponderings about eternity the mundane chore of organizing their literary archives to protect any of life's secrets that seem worth the effort. That task involves choosing what diaries, letters, drafts, and laundry lists to donate to a university or to leave in a closet for legions of biographical ragpickers to quote, misquote, or variously interpret in as yet unimaginable contexts—or to burn.

Many well-known figures contemplating their posthumous selves have been foiled in exercising control over their literary remains. Purposefully confounding future biographers, Sigmund Freud burned his early papers and admonished his wife Martha to destroy their love letters. Instead, she bequeathed us this charming insight into the youthful exuberance of the patriarch of psychoanalysis, written in 1884: “Woe to you, my Princess, when I come. I will kiss you quite red and feed you till you are plump. And if you are forward, you shall see who is stronger, a gentle little girl who doesn't eat enough or a big wild man who has cocaine in his body” [1].

Anaïs Nin, whose voluminous diaries recorded her daily life in exquisite, compulsively recorded detail, had better luck in choreographing her literary afterlife. While alive, she published volumes of carefully edited literary diaries. When someone at a seminar remarked to her that her life seemed more, well, racy than those diaries revealed, she smiled mysteriously and said that after the death of all concerned, “unexpurgated” editions would be published. Several decades later, companion volumes to the literary diaries revealed passionate incest with her father, Joachim Nin, an affair with her analyst, Otto Rank, and successfully bigamous marriages in New York and California.

When André Gide revealed that Oscar Wilde had had sexual relations with a young Arab boy in Egypt, Wilde's friend Robert Sherard lamented: “Heavens! The task of shooing hyenas away from the graves of the illustrious dead.” Sherard meant Wilde's literary grave—but what about actual graves? What about history's corpus delicti?

## The Line between Scientist and Grave Robber

How many giants and tyrants unlucky enough to have left body parts or ashes behind when they shuffled off the mortal coil could have imagined what scientists and medical practitioners of the future would do with their physical remains? Here, the line between the scientist and the grave robber blurs, as corpses are exhumed and cremation urns raided to provide organic remnants for any number of curious purposes.

Ethical debates about the appropriate care and maintenance of biological relics often begin at the autopsy table. Having removed Albert Einstein's brain, pathologist Thomas Harvey chopped it into 240 pieces and stored it in a cookie jar in his basement, often shipping slabs (mailed in mayonnaise jars) to brain researchers eager to count glia and neurons. Forty years later, Harvey lugged what remained of the brain cross-country to deliver it to Evelyn Einstein, a woman rumored to be the physicist's daughter from an affair with a New York dancer. Dr. Charles Boyd had tried to prove this paternity with his brain-chunk, but Einstein's DNA proved “too denatured to decipher.”

Harvey's volunteer driver, Michael Paterniti, described getting his hands in the cookie jar: “I actually feel as if I might puke. The pieces are sealed in celloidin—the pinkish, liver-colored blobs of brain rimmed by gold wax. I pick some out of the plastic container and hand a few to Evelyn. They feel squishy, weigh about the same as very light beach stones. We hold them up like jewelers, marveling at how they seem less like a brain than—what?—some kind of snack food, some kind of energy chunk for genius triathletes” [2].

Pilferers cannot resist snipping body parts. While Einstein was being autopsied, his ophthalmologist, Dr. Henry Abrams, dropped by and filched Einstein's brown eyes as a keepsake, storing them in a jar in a Philadelphia bank vault. There were rumors that singer Michael Jackson, a collector of body parts, offered Abrams several million dollars for the eyes.

Does confidentiality extend beyond the grave?

Beethoven's ears were hacked out and soon went missing. René Descartes's middle finger was stolen. (His head was also separated from his body for shipping—a philosopher's in-joke, since Descartes introduced the mind/ body split into Western philosophy.) Napoleon's reputed penis went on a picaresque odyssey of its own, being displayed at the Museum of French Art in New York, auctioned, and finally ending up in the possession of a urologist—or so the story goes. Josef Haydn's head was stolen by phrenologists at his burial.

In 2004, Dr. Anunciada Colon presided over the opening of a golden trunk from the 16th century, containing ashes and bone fragments presumed to belong to her ancestor Christopher Columbus, an event chronicled by a television crew. Officials at the Seville Cathedral allowed researchers at the University of Granada to borrow the bones for a DNA study. Being unsuccessful at extracting DNA from pulverized fragments, Professor José A. Lorente loaded the bones in a shoulder bag and flew them to Dallas, Texas, where more sophisticated DNA tests (developed for the victims of the terrorist attack of 9/11) provided a disappointingly short and impure sequence of mitochondrial DNA. Remaining ashes and shards were inelegantly deposited on a metal storage shelf in a lab, in a Styrofoam picnic basket labeled “Colon” in black marker, awaiting better tests [3].

Vladimir Ilyich Lenin remains the most visible deceased person. His body, or what remains of it since his brain and other organs were removed, has been viewed by the millions who have passed by his open casket in a mausoleum on Moscow's Red Square. A waterproof suit under his uniform holds in the embalming fluid. His hands and head are bathed frequently. His microtomed (31,000 sections) and dyed brain resides down the street from his body at the Moscow Brain Institute, joining the brains of his countrymen Stalin and Tchaikovsky. Many Russians who find Lenin's public resting place a macabre embarrassment think his soul will only rest (and theirs with it) once he goes underground. But who can decree his burial?

When I was four, my mother found me exhuming a goldfish we had ceremoniously buried in the garden in a little fish coffin a few days before. How different, I wonder now, was my childish curiosity and wonderment at the mysterious process happening to my no-longer-swimming fish below the earth from that of grown-up exhumers? Consider Gira Fornaciari, who unearthed 49 members of the Medici family to confirm various causes of death, or the committee that had Beethoven and Schubert dug up to transfer them to more secure zinc coffins (borrowing both heads for a bit more measuring, and swiping Schubert's luxuriant, larvae-laden hair while they were at it). Archaeologists have braved curses and biohazards to retrieve mummies from pyramids. Doctors from Japan, however, were not allowed to take DNA from King Tut's mummy to sort out his genealogy; the Egyptian government's supreme council of antiquities, after first agreeing, reversed the decision. A non-invasive x-ray of the mummy suggests a murder plot: King Tut may have been done in by a blow to the back of the skull.

## Guidelines for Bioethical Research

When a committee was convened to decide whether specimens of Lincoln's blood and bones should be tested for DNA to discover whether he suffered from Marfan syndrome, ethicists voted yes but scientists vetoed the plan, claiming that the precious material should not be destroyed in case future tests would prove more effective [4,5]. But what if they were even asking the wrong question? Lincoln once told his biographer and friend William Herndon that he had been infected with syphilis by a prostitute in Beardstown around 1835 [6]. What if a future test could prove that Lincoln had spoken the truth? Imagine, if you will, a press release from the Armed Forces Institute of Pathology revealing that hot potato about the most beloved of American presidents.

The Lincoln testing question spurred bioethicist Lori Andrews and her colleagues at the Chicago Historical Society to join with the Illinois Institute of Technology to review existing ethical issues of biohistorical research. Their conclusion, after studying professional codes from 23 other organizations: none contained guidelines for conducting biohistorical research and analysis [7]. They recommend genetic testing for “historically significant” questions. But who is to define that loaded phrase?

The newly dead are warm, soft, and somehow still human; by contrast, aged corpses and skeletons rising from the cold ground are the stuff of horror films, vampires and ghouls. While fascinating, they also unnerve. Medical examiners in fiction (Kay Scarpetta) and television (Dr. Quincy, Jordan Cavanaugh) capture wide audiences with their gruesome and graphic dissection of putrefied, maggot-ridden corpses, all in the service of solving some medical mystery.

## Respect for the Dead

Does confidentiality extend beyond the grave? Should doctors publish articles in medical journals about diagnoses that were confidential when the patient was alive? Physicians have often raced to put pen to paper and reveal the signs and symptoms of their more illustrious deceased patients. According to Anne Sexton's biographer Diane Wood Middlebrook, who used tapes of hundreds of hours of therapy sessions given to her by Sexton's therapist Dr. Martin Orne, the dead have no rights [8]. Although Dr. Orne insisted that Sexton had given him permission to do what he thought appropriate with the tapes, his colleagues howled that he had made a travesty of doctor-patient confidentiality, Sexton's wishes be damned.

The long-dead are latecomers to the game of lobbying for rights. Who owns their bones? Who is to choose the right test, the right time, the appropriate question to ask? Who gets to decide whether they should be sliced, diced, dyed, pulverized, displayed, x-rayed, photographed, and subjected to the esoteric tests developed for forensic laboratories to reveal secrets they carefully took to their graves or urns? An interdisciplinary committee? The law? The government? Should such decisions be made by bioethicists, scientists, medical examiners, lawyers, archaeologists, descendants of the deceased? Where does simple respect for the dead play into this issue?

The answers change over time and from place to place. The quagmire of ethical, legal, moral, and even aesthetic questions that surround the use (and misuse) of leftover body parts can only become more complex and contentious, not less.

A word of warning, then, to the famous not-yet-deceased: consider the disposition of your physical remains as carefully as you consider the packaging of your archive.

Swear your doctor to posthumous secrecy.

Be cremated.

And have your ashes scattered to the wind.

**Figure:**
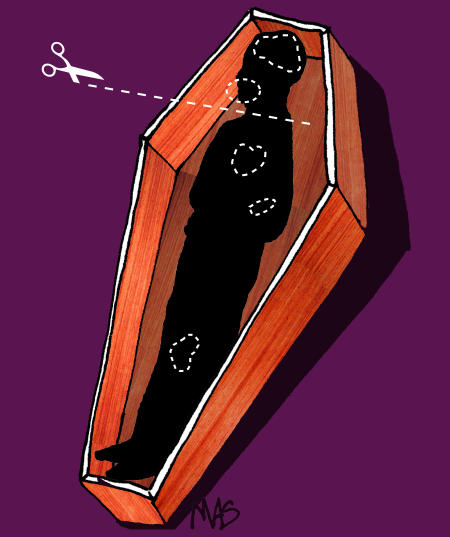
Is it ethical to remove body parts to make a tissue diagnosis? (Illustration: Margaret Shear, Public Library of Science)

**Figure:**
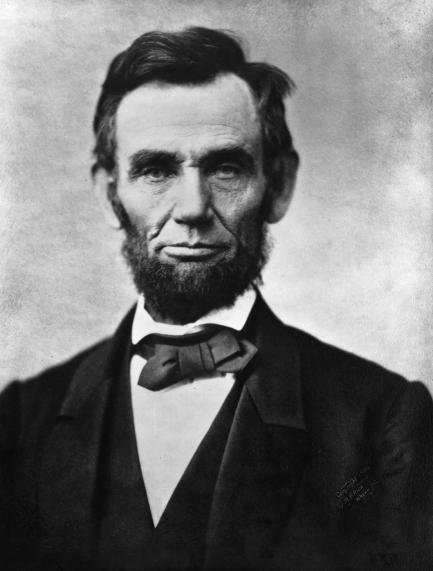
Victor McKusick of the Johns Hopkins School of Medicine chaired a committee to decide whether specimens of Lincoln's blood and bones should be tested for Marfan syndrome (Photo: Alexander Gardner, Library of Congress)
